# Supplementary material for: Diversity of Epithelial-Mesenchymal Phenotypes in Circulating Tumour Cells from Prostate Cancer Patient-Derived Xenograft Models
Source: Cancers (Basel). 2021 Jun 1;13(11):2750. doi: 10.3390/cancers13112750 (PMC8198708; doi:10.3390/cancers13112750)
Supplement: Supplementary file 1 [file cancers-13-02750-s001.zip › cancers-1115676-supplementary.pdf]

cancers

MDPI

Hassan et al Supplementary data

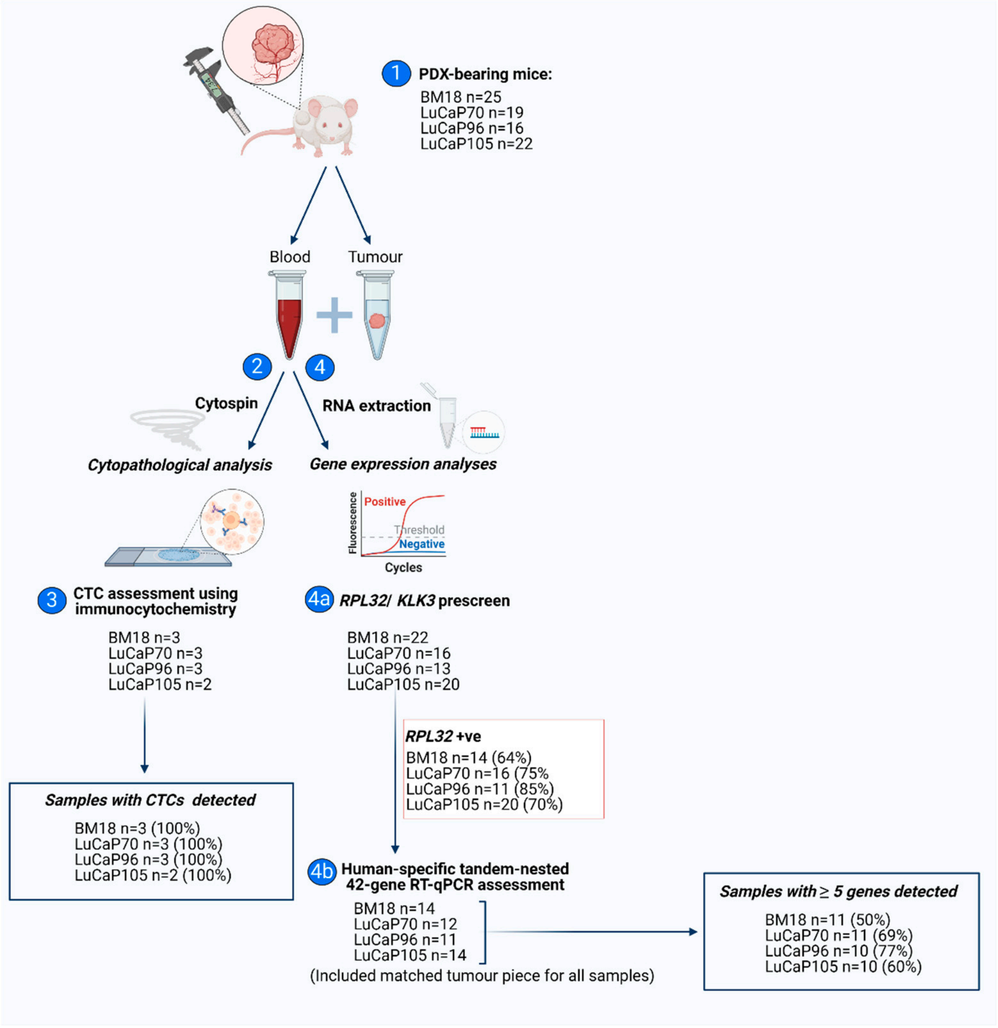

**Figure S1.** Schematic of experimental workflow, including number of samples at each stage. This study comprised of 4 prostate cancer PDX models (BM18, LuCaP70, LuCaP96, LuCaP105) that were grown as subcutaneous tumours in male severe combined immune-deficient (SCID) mice (1). Blood was collected at terminal draw using cardiac puncture. Matched tumours were collected and stored in RNAlater™ Stabilization Solution overnight and then stored briefly at -80°C until further processing. Following red blood cell (RBC) lysis, blood samples were used for either cytopathological (2) or gene expression (4) analyses. For cytopathological analysis (3), samples were cytospun onto slides and then stained for VIM, KRT, KLK3 and SERPINE1. For gene expression analysis, RNA was extracted from blood and tumour samples and assessed using a 42-gene human-specific tandem-nested RT-qPCR approach. Prior to complete 42-gene analysis, RNA extracted from blood samples was screened for CTCs using RPL32/KLK3 expression (4a). RPL32-positive blood samples and their paired tumour tissues were then assessed for the remainder of the 42 gene panel using a tandem-nested RT-qPCR assay (4b). Samples with less than 5 genes detected were excluded from further analysis. *n* denotes number of samples. CTC circulating tumour cell; SCID severe combined immune-deficient; PDX patient derived xenograft.

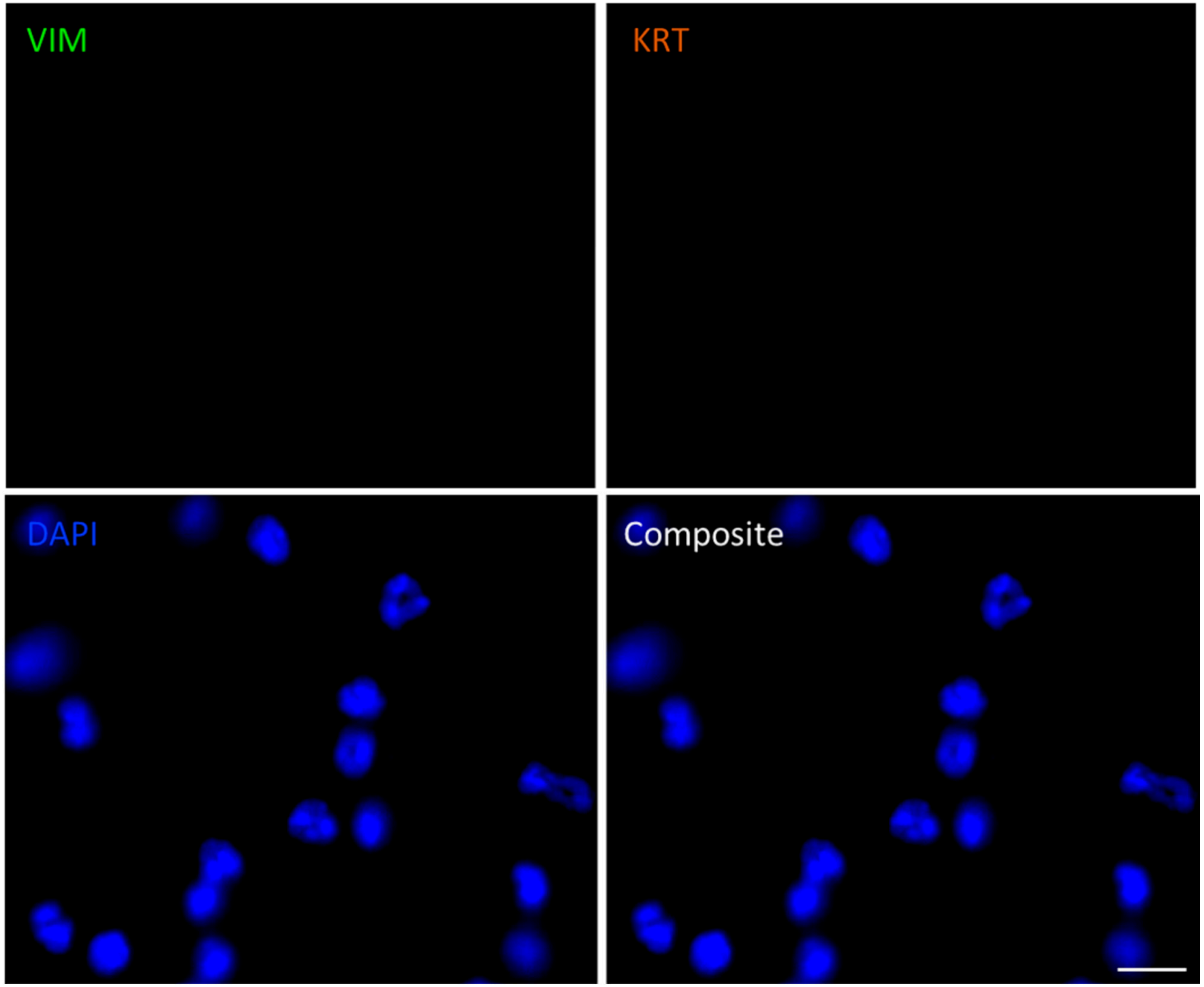


**Figure S2.** Representative images for immunofluorescent staining for VIM and KRT in negative control sample. Negative staining was observed for both VIM and KRT. Scale bar denotes 5 μm.

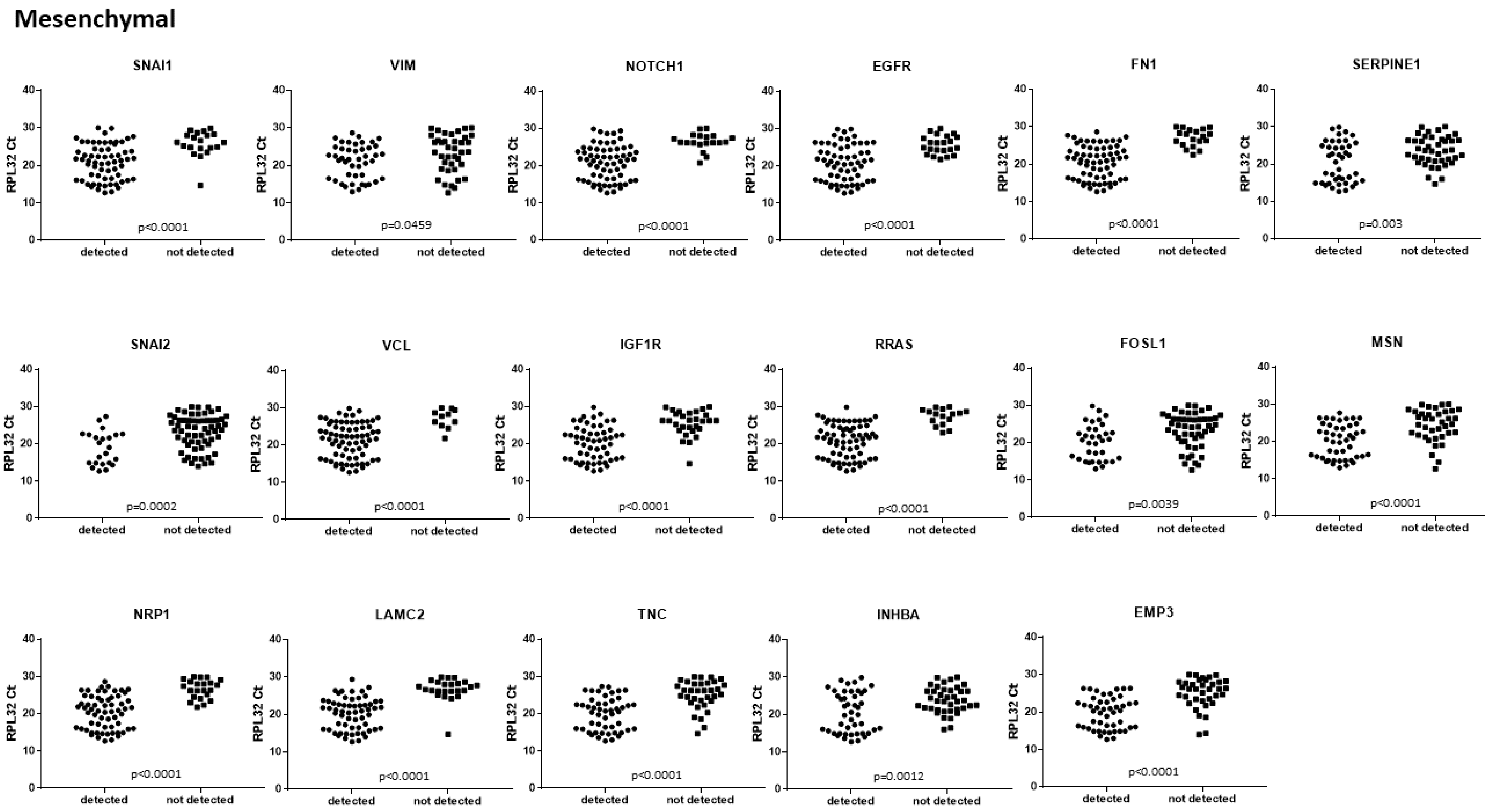
Mesenchymal
SNAI1
p<0.0001
VIM
p=0.0459
NOTCH1
p<0.0001
EGFR
p<0.0001
FN1
p<0.0001
SERPINE1
p=0.003
SNAI2
p=0.0002
VCL
p<0.0001
IGF1R
p<0.0001
RRAS
p<0.0001
FOSL1
p=0.0039
MSN
p<0.0001
NRP1
p<0.0001
LAMC2
p<0.0001
TNC
p<0.0001
INHBA
p=0.0012
EMP3
p<0.0001
RPL32 Ct
detected
not detected

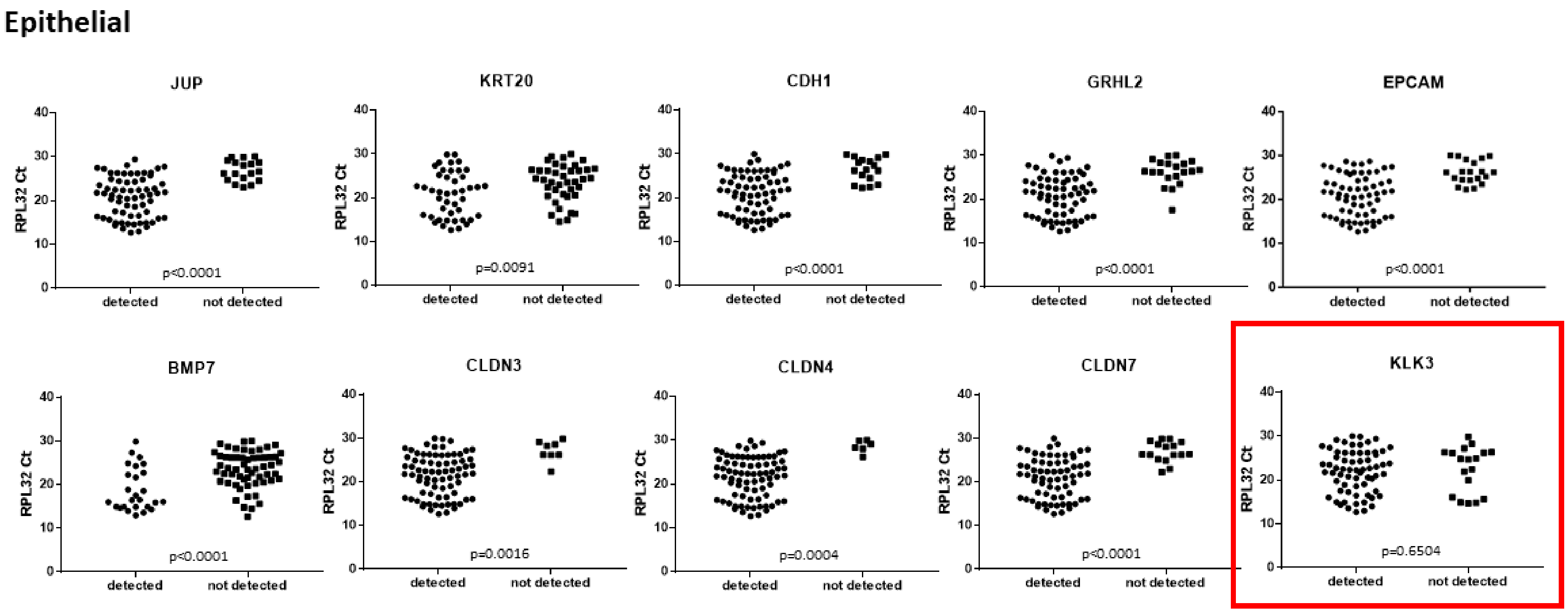
Epithelial
JUP
RPL32 Ct
p<0.0001
detected
not detected
KRT20
p=0.0091
CDH1
p<0.0001
GRHL2
p<0.0001
EPCAM
p<0.0001
BMP7
p<0.0001
CLDN3
p=0.0016
CLDN4
p=0.0004
CLDN7
p<0.0001
KLK3
p=0.6504

## Other

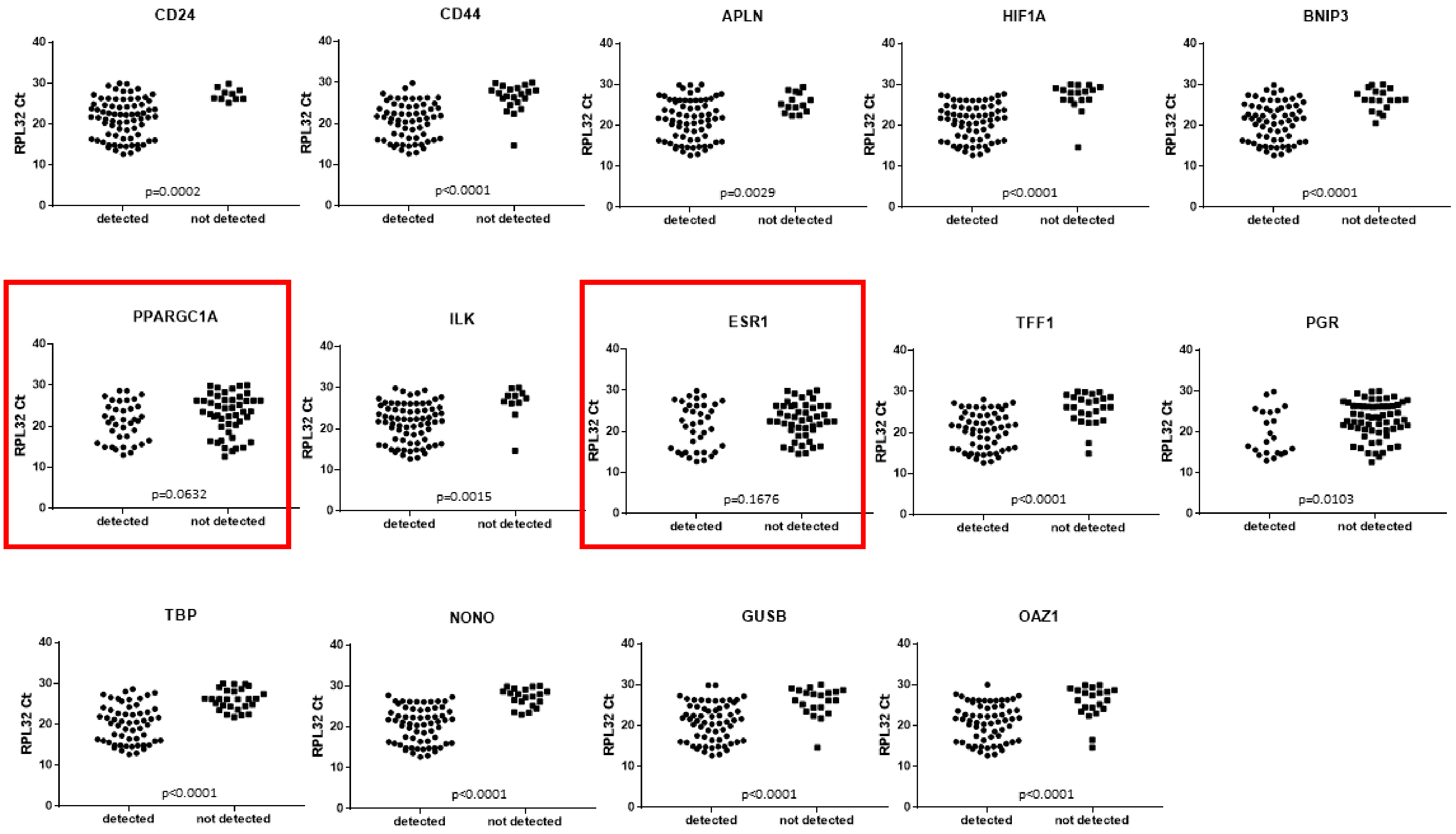


**Figure S3.** Gene expression in the context of *RPL32* expression. Each circle/square represents an individual CTC sample from a different mouse. Statistical significance determined using an unpaired t-test. $p < 0.05$ was considered significant.

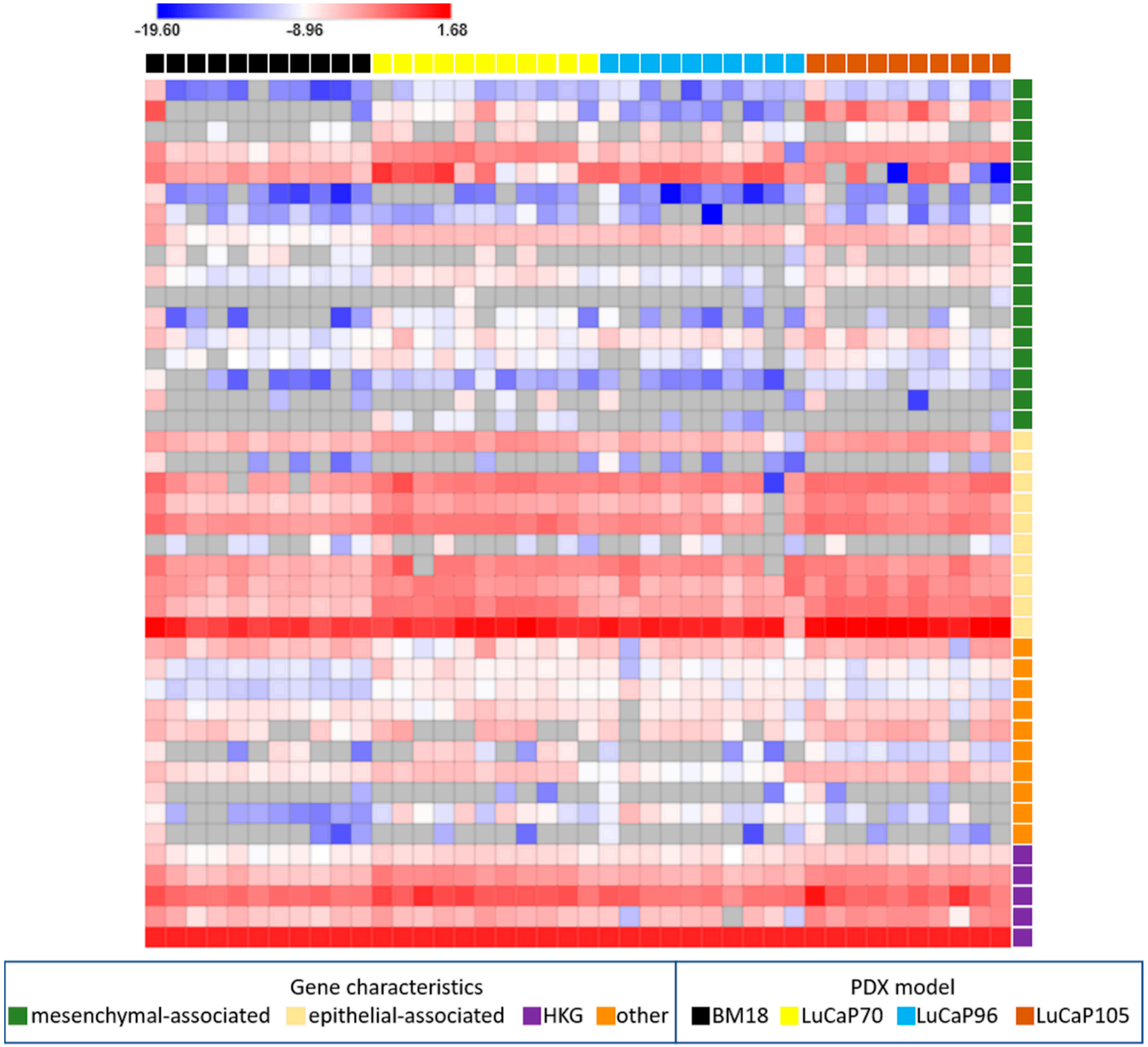


**Figure S4.** Heat map showing 42 gene panel expression profiles in tumour samples from 4 PDX models as Ct values normalised to RPL32, with global normalisation. For each sample, the PDX model is shown above the heat map. Genes are categorised as mesenchymal-associated, epithelial-associated, potential HKG (GUSB, TBP, OAZ1 and NONO), and others

(which includes HR (ESR1, PGR and TFF1), CSC (CD24 and CD44), hypoxia (APLN, HIF1A and BNIP3), metabolism (PPARGCIA), and anoikis (ILK) - associated genes). HR hormone receptor; CSC cancer stem cell; HKG housekeeper gene.

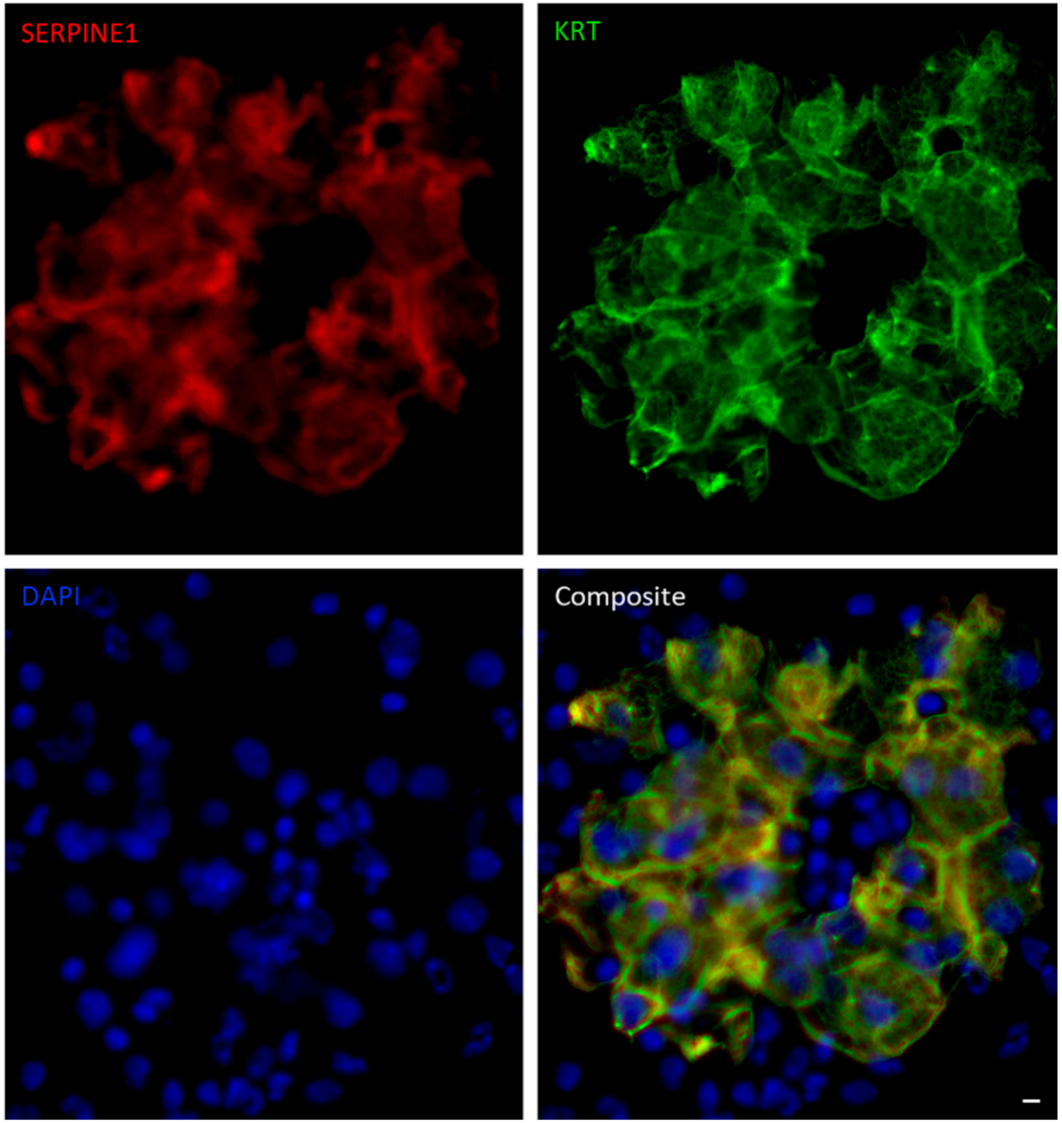


**Figure S5.** Representative images for immunofluorescent staining for SERPINE1 and KRT in negative control sample. Negative staining was observed for both SERPINE1 and KRT. Scale bar denotes 5 μm.

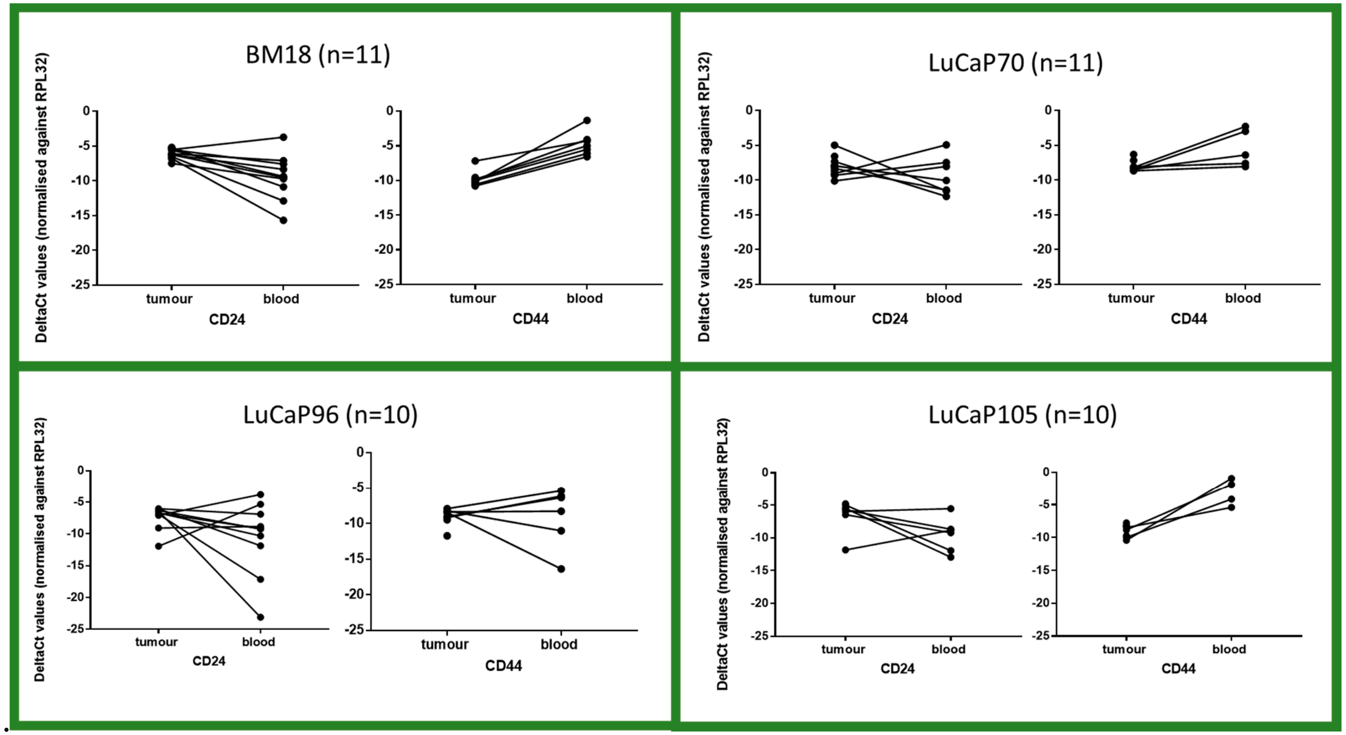


**Figure S6:** Gene expression for CSC markers, *CD24* and *CD44*, normalised to housekeeper gene *RPL32*. Downregulation of *CD24* and upregulation of *CD44* was observed in BM18 and LuCaP105 while only upregulation of *CD44* was observed in CTCs in LuCaP70. There was no change in expression in the LuCaP96 model.

**Table S1.** Summary of RT-qPCR results for *RPL32* gene in 4T1 (murine) and MDA-MB-231 (human) cell line.

| **sample** | **ct value** |
|---|---|
| 4T1 cell line (murine) | not detected |
| MDA-MB-231 cell line (human) | 34.14 |
| no template control | not detected |

**Table 2.** Summary of the estimated number of CTCs present in total mouse blood and compared to tumour weight for each mouse in this study.

| PDX ID | tumour weight (g) | estimated CTCs (total number per mouse) |
|---|---|---|
| BM18: 18-142 | 1.07 | 19 |
| BM18: 19-14 | 3.05 | 174 |
| BM18: 18-133 | 1.89 | 52 |
| BM18: 19-15 | 2.42 | 269 |
| BM18: 17-174 | 0.69 | 21 |
| BM18: 18-162 | 0.98 | 3 |
| BM18: 19-37 | 1.91 | 296 |
| BM18: 17-147 | 0.66 | 14 |
| BM18: 19-30 | 1.37 | 246 |
| BM18: 18-193 | 2.1 | 5 |
| BM18: 18-254 | 1.69 | 7 |
| LuCaP96: 18-99 | 1.42 | 4758 |
| LuCaP96: 18-192 | 1.19 | 15 |
| LuCaP96: 18-257 | 0.99 | 5 |
| LuCaP96: 19-93 | 1.34 | 85 |
| LuCaP96: 17-132 | 0.8 | 31 |
| LuCaP96: 18-138 | 1.44 | 147 |
| LuCaP96: 18-164 | 1.25 | 1958 |
| LuCaP96: 17-176 | 0.9 | 31 |
| LuCaP96: 17-208 | 0.64 | 12 |
| LuCaP96: 17-175 | 0.9 | 2 |
| LuCaP70: 19-101 | 0.98 | 16 |
| LuCaP70: 18-214 | 0.48 | 1 |
| LuCaP70: 19-42 | 0.59 | 49 |
| LuCaP70: 17-146 | 1.16 | 3 |
| LuCaP70: 17-182 | 0.92 | 29 |
| LuCaP70: 17-140 | 1.19 | 137 |
| LuCaP70: 19-50 | 0.21 | 2 |
| LuCaP70: 18-159 | 0.14 | 278 |
| LuCaP70: 18-261 | 1.33 | 277 |
| LuCaP70: 18-238 | 1.1 | 4 |

| | | |
|---|---|---|
| LuCaP70: 19-133 | 0.97 | 3 |
| LuCaP105: 19-39 | 0.91 | 13 |
| LuCaP105: 18-179 | 0.88 | 308 |
| LuCaP105: 19-90 | 0.31 | 3 |
| LuCaP105: 17-158 | 1.07 | 2 |
| LuCaP105: 19-121 | 0.67 | 2 |
| LuCaP105: 19-41 | 1.26 | 4 |
| LuCaP105: 17-165 | 0.87 | 7 |
| LuCaP105: 17-166 | 0.84 | 4 |
| LuCaP105: 18-265 | 1.31 | 183 |
| LuCaP105: 19-60 | 0.9 | 45 |

**Table S3:** List of genes analysed in the study and the primer sequences used for qRT-PCR analysis.

| Gene Name | RT primer name | RT Sequence | 5' primer name | 5' Sequence | 3' primer name | 3' Sequence | 5 i | 5' Sequence | 3 i | 3' Sequence |
|---|---|---|---|---|---|---|---|---|---|---|
| Hs RPL32 | Hs L 32 RT | CAGAAAACGTGCACATGAGCTGC | 5o' Hs L 32 | CAGGGTTCGTAGAA-GATTCAAGGG | 3o' Hs L 32 | CTTGGAGGAAACATT-GTGAGCGATC | 5 i Hs L 32 | GATCTT-GATGCCCAACATT-GGTTATG | 3 i Hs L 32 | GCACTTCCAGCTCCTTGACG |
| Hs CD24 | Hs CD24 RTb | GGGCGACAAAGTGA-GACTGTCTAAAA | 5o' Hs CD24 | CTGTTCTCTTGG-GAACTGAACTCACTTT | 3o' Hs CD24 | GTCGTGGTCAATGCAATTCTACTCTTT | 5 i Hs CD24 | CTGAGCAACTCTTGAT-TATCCATATTGAGTCAAA | 3 i Hs CD24 | CTCTTGTT-GAATGGAAACAGGTGA-TAGGAAA |
| Hs VIM | Hs VIM RT | CTAAATCTTGTAGGAG-TGTCGGTTGTT | 5o' Hs VIM | CTAGA-GATGGACAGGTTATCAACGAA | 3' Hs VIM | CTTGTAGGAG-TGTCGGTTGTTAAGAA | 5 i Hs VIM | GCACACACTCAGTGCAG-CAATATAT | 3 i Hs VIM | GCAGAAAGGCACTTGAAA-GCTGTTTCTT |
| Hs CDH1 | Hs CDH1 RT | CTCTGTCTTTGGCTGCAGCAC-TTTA | 5o' Hs CDH1 | CTGTGCCCAGCCTCCATGTTTT | 3o' Hs CDH1 | CTGGATAGCTGCCCATT-GCAAGTTA | 5 i Hs CDH1 | CAACTCTCAC-TCCTGAATTCAGTTGCTTT | 3 i Hs CDH1 | CAAGATGTGGCCAGA-CAAAGACACAAA |
| Hs ILK | Hs ILK RTb | GCTGGGGTAGTACCATGACTG | 5o' Hs ILK | GATGCAG-GACAAGTAGGACTG-GAA | 3o' Hs ILK | CAAC-CAGAGGCCTGCTGCTTT | 5 i Hs ILK | CTCCAGAGGTGTCGG-GACA | 3 i Hs ILK | GGGAGGTGCATTCCCCCAA |
| Hs EGFR | Hs EGFR RTb | CAGTTCCTGTGGATCCAGAGGAG-GAGTATGT | 5o' Hs EGFR | GGACTGAAGGAGCTGCCCATGAGAAAT | 3o' Hs EGFR | CTTGCAC-GTGGCTTCGTCTCG-GAATTT | 5 i Hs EGFR | CAGTGGCGG-GACATAGTCAGCAGTGA | 3 i Hs EGFR | CCCATTGGGACAGCTT-GGATCACACTTTT |
| Hs CD44 | Hs CD44 RTb | CAACACATAGTGGCCTAATGTCCAGTTTCTTT | 5o' Hs CD44 | GGTATCTCCTTTCTGAGGCTCCTACTAAAA | 3o' Hs CD44 | GCAGAGGCTGGGAA-TAGTGCAGAAA | 5 i Hs CD44 | CAGGCAATGCTTCTCAGACCACAAA | 3 i Hs CD44 | CTCTGTCTAA-GCCCAGCCCTGATTTA |
| Hs ESR1 | Hs ESR1 RT | CCAGGGCCACGCTGG-GAAATGAA | 5o' Hs ESR1 | GTTCCAGTGGGCACTG-TACTTGGATCTT | 3o' Hs ESR1 | CAGCTCCATGCCCCAGGGCTAAAT | 5 i Hs ESR1 | GTGCCTTACACAGGGGTGAACTGTT | 3 i Hs ESR1 | GCCCCTGCTCCTTTCAAC-TACCATTT |
| Hs PGR | Hs PGR RT | GGGAGAGATCTCACAAAGTAG-GAAGCAAA | 5o' Hs PGR | GGAG-GAGGGAGGTATCAATTCACATACCTTT | 3o' Hs PGR | GGCAGGTAAACTCTAG-GAGAAGGCAAATT | 5 i Hs PGR | CGA-GACTCAGCCAAATGTCATTTCTGTAA | 3 i Hs PGR | CTGCTTGGAA-GACTCAGGGAAGATT |

| | | | | | | | | | |
|---|---|---|---|---|---|---|---|---|---|
| Hs TFF1 | Hs TFF1 RT | GGCAGCCGAGCTCTGGGACTAAT | 5o' Hs TFF1 | CCCCCCGTGAAAGA-CAGAATTGTGGTTTT | 3o' Hs TFF1 | GGCAGATCCCTG-CAGAAGTGTCTAAA | 5 i Hs TFF1 | GTCACGCCCTCCCAG-TGTGCAA | 3 i Hs TFF1 | GTCGTCGAAACAG-CAGCCCTTAT |
| Hs EP-CAM | Hs EP-CAM RT b | GTTTTGCTCTTCTCCCAAGTTTT-GAGCCATT | 5o' Hs EPCAM | CTCGCGTTCGGGCTTCT GCTT | 3o' Hs EPCAM | CAACTGAAGTACAC-TGGCATTGACGATTATT | 5 i Hs EPCAM | CGC GGC GAC GGC GAC TTT T | 3 i Hs EPCAM | GCAGTTTACGGCCAGCTT-GTAGTTTT |
| Hs OAZ1 | Hs OAZ1 RT | GGCTTTTGGAGAG-CAATGGAGGGGATTAA | 5o' Hs OAZ1 | GGCCTACACGTTCGA-GAGAGAGTCTT | 3o' Hs OAZ1 | CTCCTCAAAGTACAAGG ATAATCTGAATCAT | 5 i Hs OAZ1 | CCTTGTCAGCCGGGTGGG-TAGGAA | 3 i Hs OAZ1 | CAGGCGAGATGAGCGAG-TCTA |
| Hs CK20 | Hs CK20 RT | GGACTCCCAAAGGGGGTTTACA ATTCAGAAA | 5o' Hs CK20 | GCTCCCTGCAA-GAAATCAGCCATAA | 3o' Hs CK20 | CAGCTTGTAATT-GATGTGCTTCATGA-TAAA | 5 i Hs CK20 | CGGGAATCC-TATTTATCAGACTCTG-TAATT | 3 i Hs CK20 | GGCAATTT-GCAGCTCCTCTGAGTA |
| Hs SNAI1 | Hs SNAI1 RT | CGCAGACAGGCCAGCTCAG-GAAT | 5o' Hs SNAI1 | CACATCCTTCTCAC-TGCCATGGAATT | 3o' Hs SNAI1 | GCTGCCCTCCCTCCACA GAAAT | 5 i Hs SNAI1 | GCCCCACAGGACTTT-GATGAAGACCAT | 3 i Hs SNAI1 | GAGCCCAGGCAGAG-GACACAGAA |
| Hs NOTCH 1 | Hs NOTCH 1 RT | GCAAGTGCCACAG-TCCACACATCTCAT | 5o' Hs NOTCH 1 | GCATCAC-CTGCCTGTTAGGAGAA | 3o' Hs NOTCH 1 | GGCATACACACTCCGA-GAACACATTT | 5 i Hs NOTCH 1 | GCACGCGGATTAATTTG CATCTGAAA | 3 i Hs NOTCH 1 | GGCAACATCTAACCCATATG CTTTCACTT |
| Hs CLDN3 | Hs CLDN3 RT | CGTGCTCCAGAAGGGTGAGGTTT CA | 5o' Hs CLDN3 | GCCCACCCCCAGAA-GCCAGGAA | 3o' Hs CLDN3 | GGTT-GGTCCCTGGGCCCCGAA GT | 5 i Hs CLDN3 | CGCTGGACTGGGGCAGCTT | 3 i Hs CLDN3 | CGACTGCCCGGCCCGCAAA |
| Hs CLDN4 | Hs CLDN4 RT | GCAAGGCACAG-TCCCAGAGGTGATAT | 5o' Hs CLDN4 | GGCCAGGA-TAGCTTAACCCTGACTT T | 3o' Hs CLDN4 | CAAGGCCTACCCG-GAACAGAGGAGAT | 5 i Hs CLDN4 | GGCGTTGGCCAC-TGTCCCCATTTA | 3 i Hs CLDN4 | GCAGGCAGACAGAGTGGG-GAAAAT |
| Hs CLDN7 | Hs CLDN7 RT | CCCTTTCAGGCATCTAGACAC-TCCCATA | 5o' Hs CLDN7 | GCCTTGGTAGCTT-GCTCCTGGTAT | 3o' Hs CLDN7 | GTCAGGCTGGGG-CAAGGAGAT | 5 i Hs CLDN7 | CCCTTTGATCCCTAC-CAACATTAAGTATGA | 3 i Hs CLDN7 | CGGTACCCAGCCTT-GCTCTCATT |
| Hs FOSL1 | Hs FOSL1 RT | CTGGTGCCACTGG-TACTGCCTGTGT | 5o' Hs FOSL1 | GAGTCAGAACCCAG-CAGCCGTGTA | 3o' Hs FOSL1 | GTCAGAGGCCTGGGG-TAACTGCT | 5i' Hs FOSL1 | GCAGGCAGCCCAG-CAGAAGTT | 3i' Hs FOSL1 | GCAGCTCCTGACTGCCAC-TCAT |

| | | | | | | | | | |
|---|---|---|---|---|---|---|---|---|---|
| Hs PPARG C1A | Hs PPARG C1A RT | CCTGCCAATCAGAGGAGA-CATCTTTATTTT | 5o' Hs PPARG C1A | CAGTGAGTCTTCTGG-TACACAAGGCAATAA | 3o' Hs PPARG C1A | TGACTGGCAA-TAGTCATGGTCAC-CAAACA | 5i' Hs PPARG C1A | CAGCAAGTCCTCAG-TCCTCACTGGT | 3i' Hs PPARG C1A | GACTGGGCCGCTT-GGTCTTCCTTT |
| Hs VCL | Hs VCL RT | CATGGGCTAGAAACCCAG-TCTGCCTATT | 5o' Hs VCL | CTGAGCCTGGCTGG-CACAGAAA | 3o' Hs VCL | GATAGAGGGTG-CACAAGTG-TAACTGTGTTT | 5i' Hs VCL | GTCCCAG-GAGCTGCCCAGAGTT | 3i' Hs VCL | GAA-GAGGCCCCCATTCCTTTCTGATGT |
| Hs IGF1R | Hs IGF1R RT | CTGGAT-TCCTGGCCGAGGGAAAGT | 5o' Hs IGF1R | CGACTGACCTGTCTTT-GGAACCAGAACAT | 3o' Hs IGF1R | CCTGGTCCCCCAGCAA-GAAGCAAA | 5i' Hs IGF1R | CTCCTTCGCACTGGCGTT-GAGT | 3i' Hs IGF1R | GCGTCTGTGCTGCCTCCAC-CTA |
| Hs RRAS | Hs RRAS RT | GCACACAGTGGCAG-TAGCCCAGAA | 5o' Hs RRAS | GTAGCCCAGGCAAGA-GAGAAGCAA | 3o' Hs RRAS | GGCCAG-GAAGTAAGGGTGGG-TATGTGA | 5i' Hs RRAS | CGCACCTTGCTGTGTGAC-CTGA | 3i' Hs RRAS | GTCCTGGGA-GACCCAGATGAGGAAATT |
| Hs GRHL2 | Hs GRHL2 RT | CTGGTTTCACAG-GAGCCAGGTATCTT | 5o' Hs GRHL2 | GCTGCCATGTGA-GAGCTGTGAACA | 3o' Hs GRHL2 | GGCACATTCTTTCTCAC-TCACTTCCTTAAA | 5i' Hs GRHL2 | CCT CCT GGG CCA CCA GAT GGA AA | 3i' Hs GRHL2 | CAGCATCCAGGAG-TGAGGCAAACAATA |
| Hs EMP 3 | Hs EMP 3 RT | CTGCAGGCCCATGTTTT-GGTGTCGT | 5o' Hs EMP 3 | GCCCGAGCGAGGGACAAGACT | 3o' Hs EMP 3 | GTCCAAAGTGGCCAC-GAAAAGCAGTAT | 5i' Hs EMP 3 | GACTCCGACTCCAGCTCTGACTTTTTT | 3i' Hs EMP 3 | GAGGAGTGACATGGCTG-CAGTGGAA |
| Hs MSN | Hs MSN RT | GAGCCTACTGGCATCAAGCTCTA-GAT | 5o' Hs MSN | CCAGCCCCATCAG-GACTCTTGTGAAAA | 3o' Hs MSN | GATGGCTGTGGAGCAG-GAGCTAT | 5i' Hs MSN | GAGGATATGTTCACAC-CTAGCGTCAGTATT | 3i' Hs MSN | GTAGCTCTGAGAGGGGAA-GAGACCTAAAA |
| Hs TBP | Hs TBP RT | CAC-TTAAAGTTTTCTCCCTCAAAC-CAACTTGT | 5o' Hs TBP | GGTGGTGTTGTGAGAA-GATGGATGTTGAGTT | 3o' Hs TBP | CTGCCCAGATAGCAG-CACGGTAT | 5i' Hs TBP | GGCAC-CAGGTGATGCCCTTCTG-TAA | 3i' Hs TBP | CGCGGTGTTCTCAGTG-CACAAATAAT |
| Hs GUSB | Hs GUSB RT | GTGTTCCCTGCTAGAA-TAGATGACCACAAAA | 5o' Hs GUSB | GATACTGGAA-GATTGCCAATGAAAC-CAGGTAT | 3o' Hs GUSB | CGTGAACAGTCCAG-GAGGCACTTGTT | 5i' Hs GUSB | CCCACTCAG-TAGCCAAGTCACAATGTTT | 3i' Hs GUSB | GCAGGTGGTATCAGTCTT-GCTCAAGTAAA |
| Hs NONO | NONO RT | GAGTGG-CATATTGTCCAGCTCCACTTT | 5o' Hs NONO | GACCTTCACCCAAC-GAAGCCGTCTTTT | 3o' Hs NONO | CTAGGGTTCGGGTTTCCAAGCGGATAAA | 5i' Hs NONO | CCTCCCGACATCACTGAG-GAAGAAAT | 3i' Hs NONO | GAA-GACTTCGCCTGCCTTTCCATATTT |
| Hs FN1 | Hs FN1 RT | GAACGTCCTGCCATT-GTAGGTGAATGGTAA | 5o' Hs FN1 | CTAGAAA-TAGATGCAACGATCAG-GACACAA | 3o' Hs FN1 | GCGGTTGG-TAAACAGCTGCACGAA | 5i' Hs FN1 | GGAAGTGTGA-GAGGCACACCTCTGT | 3i' Hs FN1 | GGGGCCAGATCCGCTCGATGT |

| | | | | | | | | | |
|---|---|---|---|---|---|---|---|---|---|
| Hs HIF1A | Hs HIF1A RT | CTACCACGTACTGCTGGCAAA-GCATTAT | 5o' Hs HIF1A | GCTACAATACTG-CACAAACTTGGTTAG-TTCAA | 3o' Hs HIF1A | CGAAAAAGGGA-TAAACTCCCTAGCCAAAAA | 5i' Hs HIF1A | GCTGGATCACAGA-CAGCTCATTTTCT | 3i' Hs HIF1A | GCTACTG-CAATGCAATGGTTTAAATAC-CAAAAA |
| Hs NRP1 | Hs NRP1 RT | GAGCCTGAATCAGCCATTCG-CATTTT | 5o' Hs NRP1 | GGCATT-GCCTCGCTGCTTTCTT | 3o' Hs NRP1 | CAGGCAGACGCGGGA-GAACGA | 5i' Hs NRP1 | CTCCAAGACGGGCTGAG-GATTGTACA | 3i' Hs NRP1 | GAA-GAGCCCCAACTCCGCCTAGA |
| Hs SNAI2 | Hs SNAI2 RT | CATGCGCCAGGAATGTTCAAA-GCTAAT | 5o' Hs SNAI2 | CTGATGGCTAGATTGA-GAGAATAAAAGACAG-TAA | 3o' Hs SNAI2 | CTCCTCCCCCAAGGCACATACTGTTAAT | 5i' Hs SNAI2 | GAAGAGATCTGCCAGAC-GCGAACT | 3i' Hs SNAI | CAATGGCAACCAGACAAC-CGACATGTAAT |
| Hs BMP7 | Hs BMP7 RT | GCTAGGTTTTGCCTGCACAGCTT-GTA | 5o' Hs BMP7 | GAATTCAGACCCTTT-GGGGCCAAGTTTTT | 3o' Hs BMP7 | CTTGTTCATT-GGATGCTGCCAC-TGAAAAA | 5i' Hs BMP7 | GGAACCAGCAGAC-CAACTGCCTTTT | 3i' Hs BMP7 | GATCAAAA-GCCATATGCTGCTCATGTTT |
| Hs SER-PINE1 | Hs SER-PINE1 RT | GAGTGATGGCAATGTGACTG-GAACAGAAATA | 5o' Hs SER-PINE1 | CTTGGCCTCTCCTT-GGAGGACCTTTA | 3o' Hs SER-PINE1 | GTAAGCCCCGTAG-TTCCATCCTGCAAA | 5i' Hs SER-PINE1 | CACCTGAGACCCTGGGA-GAGAAGTTT | 3i' Hs SER-PINE1 | CGTCACCGTCTGGTTTGGA-GACCTTAA |
| Hs LAMC2 | Hs LAMC2 RT | CAGTCACACTTGGAGTCTAGCAG-TCT | 5o' Hs LAMC2 | GCATTCACTGCGA-GAAGTGCAAGAAT | 3o' Hs LAMC2 | CATCTGGCTCCTGTCACACCTGGTTTA | 5i' Hs LAMC2 | GGCACAGAGAAAGGGAC-CGCTGTTT | 3i' Hs LAMC2 | CGAGCACTAAGAGAACCTTT-GGAGTTACAA |
| Hs TNC | Hs TNC RT | GCTCTAGGGCTCTAGGG-TATCTCACTTT | 5o' Hs TNC | CCACAC-TCCAGGTACTTCTTCCAAGGA | 3o' Hs TNC | GAATTGGGGATTTAGAA-GCACAGAGTAGACTT | 5i' Hs TNC | GGT CTC TCG CCC ATC GGA AAG AAA | 3i' Hs TNC | CAAATCAGTT-GTCCCTGATCTTCTTGAAA-GAATT |
| Hs BNIP3 | Hs BNIP3 RT | CCAGGGTGATCACCTAATAATCG-GAGACTTAAT | 5o' Hs BNIP3 | CTCCACCAGCAC-CTTTTGATGAAGAACT | 3o' Hs BNIP3 | GATCCCATAGGTG-TAATTATA-GATCAACATGATTTT | 5i' Hs BNIP3 | GTTAGTGGAT-TACTTCTGAGCTT-GCAACATA | 3i' Hs BNIP3 | GTGACGTGGCCACCCCAG-GATCTAA |
| Hs APLN | Hs APLN RT | GAAGGGAAGGCCATGGAG-TCCAGTGATT | 5o' Hs APLN | CCAGCTGGGCTG-GAAGGTTCCATAA | 3o' Hs APLN | CTGGGGTTGAGCGGTAG-TCTCAGT | 5i' Hs APLN | CTGGCCAGCTGCCCCCA-TAACT | 3i' Hs APLN | GCCTGAGCCGCCCCTATTAGAGTA |
| Hs IN-HBA | Hs IN-HBA RT | GTAG-GAAAGTGCCTGTGATGGGCCCTTT | 5o' Hs INHBA | GTGAAAGAGACAG-GAATTGGGAGGGAAA | 3o' Hs INHBA | CTAGTCCACACTACTG-CAGACTAGATTG | 5i' Hs INHBA | GGGAGAATGGTG-TACCCTTTATTTCTTCTGAAAT | 3i' Hs INHBA | CAAGGGGGGAAAGGACAA-TACCCCGTTTAA |

| | | | | | | | | | | |
|---|---|---|---|---|---|---|---|---|---|---|
| Hs JUP | Hs JUP RT | CAGTTGGTCGGTGGAGTTCAG-TGAGAAAAT | 5o' Hs JUP | GCTGGGGAA-GCCGGGGTGTAACTT | 3o' Hs JUP | CTGGAGA-CAGGGAGGCTG-GAGGTTT | 5i' Hs JUP | CAGAGGTCCTC CGTAGGGTCTTTCTT | 3i' Hs JUP | CAGAATGGGTACTTGAG-TCTGAAGCTTTA |
| Hs KLK3 | KLK3 RT | GGGGGTCCAG-TCCCTCTCCTTACTTCAT | 5o' KLK3 | CCAAGACTCAA-GCCTCCCCAGTTCT | 3o' KLK3 | CAGTGCCTCCAG-GACAGAGTGGGTTAT | 5i' KLK3 | GTCCTTAGGTGTGAGGTCC AGGGTTGCT | 3i' KLK3 | CACCCCTCTTTCATCTCTG-TACCCCAC AA |
